# Different Roles of the Left and Right Ventrolateral Prefrontal Cortex in Cognitive Reappraisal: An Online Transcranial Magnetic Stimulation Study

**DOI:** 10.3389/fnhum.2022.928077

**Published:** 2022-06-10

**Authors:** Si Cheng, Xiufu Qiu, Sijin Li, Licheng Mo, Feng Xu, Dandan Zhang

**Affiliations:** ^1^Institute of Brain and Psychological Sciences, Sichuan Normal University, Chengdu, China; ^2^School of Psychology, Shenzhen University, Shenzhen, China; ^3^Shenzhen Yingchi Technology Co., Ltd., Shenzhen, China; ^4^Shenzhen-Hong Kong Institute of Brain Science, Shenzhen, China

**Keywords:** ventrolateral prefrontal cortex, emotion regulation, reappraisal, online transcranial magnetic stimulation, social exclusion, inhibition process

## Abstract

The ventrolateral prefrontal cortex (VLPFC) plays a pivotal role in cognitive reappraisal. Previous studies suggested a functional asymmetry of the bilateral VLPFC, but the evidence is still insufficient during cognitive reappraisal. In this study, we conducted an online single-pulse transcranial magnetic stimulation (spTMS) to investigate the causal and distinct roles of the left and right VLPFC in reappraisal. Participants were instructed to reappraise (down-regulate) or attend to pictures depicting social exclusion scenarios while the spTMS was applied over the left or right VLPFC of the participants’ brains. The results showed that spTMS of either the left or the right VLPFC would increase reappraisal difficulty. Meanwhile, the outcome of reappraisal (measured by self-reported negative feelings) significantly deteriorated when the right (but not the left) VLPFC was temporally interrupted by spTMS, while the verbal fluency during oral reporting of the reappraisal strategy was significantly reduced when the left VLPFC was interrupted by spTMS. Taken together, these findings provide causal evidence for the involvement of left and right VLPFC with distinct roles: while the left VLPFC is responsible for the linguistic especially semantic process of generating and selecting appraisals according to the goal of emotion regulation, the right VLPFC plays a critical role in inhibiting inappropriate negative emotions and thoughts generated by the effective scenarios. These findings deepen our understanding of the neurocognitive mechanism of emotion regulation.

## Introduction

Cognitive reappraisal (reappraisal for short) is an effective and adaptive strategy to regulate negative emotions and has long-term benefits (Erk et al., 2010; McRae and Gross, 2020). Reappraisal regulates emotions by manipulating the semantic representation of affective scenarios, which requires a series of cognitive processes, including conflict monitoring, inhibition, selective attention, and working memory (Ochsner et al., 2012; Buhle et al., 2014). Neuroimaging evidence consistently revealed that the cognitive control processes of reappraisal mainly depend on the prefrontal cortex (Buhle et al., 2014; Kohn et al., 2014), in which the ventrolateral prefrontal cortex (VLPFC) is the most distinguished prefrontal region. The VLPFC has been found to be consistently activated in voluntary emotion regulation regardless of regulatory strategies (cognitive reappraisal, distraction, expression inhibition, etc.) and regulatory goals (up- or downregulation; Ochsner et al., 2012; Kohn et al., 2014; Morawetz et al., 2017). Previous studies in our laboratory by transcranial magnetic stimulation (TMS) have further demonstrated that the VLPFC is an essential brain region in reappraisal (He et al., 2020b; Zhao J. et al., 2021; Li et al., 2022).

However, controversy remains regarding whether the left and right parts of the VLPFC play the same role during reappraisal implementation. While some studies found that the bilateral VLPFC was involved in reappraisal without any functional difference (refer to meta-analyses by Buhle et al. (2014), Kohn et al. (2014), Morawetz et al. (2016)), some other studies reported different findings. For example, Johnstone et al. (2007) found that the left VLPFC (lVLPFC) but not the right VLPFC (rVLPFC) was involved during reappraisal. On the contrary, the unilateral activation of the rVLPFC was observed by Phan et al. (2005) in studies with similar experimental designs.

The lVLPFC largely overlaps with Broca’s area; the latter is a well-known brain region for speech production and semantic/phonological processing of language (Price et al., 2011; Zhang et al., 2018; Zhao Z. et al., 2021). Thus, the lVLPFC has been consistently involved in semantic processing and intrinsic language generation (Phan et al., 2005; Nagel et al., 2008; Souza et al., 2009; Conner et al., 2019; Zhang et al., 2022). Meanwhile, evidence showed a correlation between lVLPFC activation and verbal fluency (Cho et al., 2012; Fillman et al., 2016). In addition, meta-analyses showed significant functional connectivity between the lVLPFC and linguistic brain regions, including the temporal pole and the left inferior, middle, and superior temporal gyrus (Billingsley et al., 2001; Kohn et al., 2014; He Z. et al., 2018; Banjac et al., 2021). Unlike the lVLPFC, the rVLPFC is usually considered an inhibition-related area (Aron et al., 2004). Neuroimaging studies have demonstrated that the rVLPFC plays a critical role in inhibition control and is especially active during motor inhibition (refer to meta-analysis by Levy and Wagner (2011)). It is found that the cortical thickness of the rVLPFC in childhood could predict behavior inhibition in adulthood (Sylvester et al., 2016). Meanwhile, the hyperactive rVLPFC may explain the deficits in response inhibition in schizophrenic patients (Kaladjian et al., 2007, 2011).

In line with their different cognitive roles, we hypothesized that the left and right parts of the VLPFC are responsible for different roles during cognitive reappraisal: while the rVLPFC inhibits inappropriate negative thoughts associated with a current effective scenario, the lVLPFC is critical for the semantic selection of an appropriate new explanation for the scenario according to the goal of emotion regulation. So far, as we know, there has been no study that has examined the separate roles of the left and right VLPFC in the process of cognitive reappraisal.

To test our hypothesis, this study conducted a single-pulse transcranial magnetic stimulation (spTMS) to transiently interrupt the functions of either the left or the right part of the VLPFC and examined the changes in reappraisal performance using two categories of indexes/measures. First, the self-reported negative feeling was explored to reveal the effect of interrupted lVLPFC or rVLPFC on reappraisal outcome (He Z. et al., 2018; He et al., 2020a,b; Cao et al., 2022), i.e., whether the initial negative emotion was successfully inhibited and improved by reappraisal implementation. We expected that the reappraisal outcome would be affected to the most extent when the rVLPFC was interrupted by spTMS. Meanwhile, we used the self-reported rating of reappraisal difficulty to examine the cognitive cost of cognitive reappraisal (Ortner et al., 2016; Troy et al., 2018). It is suggested that increased conflict between the initial (often negative) and the new (less emotional) appraisal would raise reappraisal difficulty because of extra paid cognitive effort (Ortner et al., 2016). We expected that the spTMS might induce extra cognitive cost for inhibition or semantic processing, resulting in enhanced reappraisal difficulty during interruption of both the left and right parts of the VLPFC. Second, we required the participants to verbalize their emotional feelings and emotion regulation strategies during the reappraisal. The recorded oral reporting was then used to calculate verbal fluency, the total number of words, and the onset time of oral reporting (also refer to Walsh (1983), Cho et al. (2012), Marini and Urgesi (2012), Urgesi et al. (2016), Fu et al. (2018)). These indexes could help to understand the effect of interrupted lVLPFC or rVLPFC on language functions, such as semantic representation and selection and language organization and production. We expected to observe significant changes in the oral indexes when the lVLPFC was temporally interrupted by spTMS.

## Materials and Methods

### Participants

Thirty-one mentally healthy, right-handed individuals (15 women, age: 19.1 ± 1.9 years old, M ± SD) were recruited from Shenzhen University (Guangdong, China). All the participants were with normal or corrected normal vision and without any psychiatric history or medicine use. Each of them filled out the trait form of Spielberger’s State-Trait Anxiety Inventory (STAI-T: 52.7 ± 2.8; Spielberger et al., 1983) and the Self-Rating Depression Scale (SDS:.4 ±.1; Zung et al., 1965) before the experiment. All the participants were mentally healthy according to their scores in STAI-T and SDS. The protocols of the study were approved by the Ethical Committee of Shenzhen University. Informed consent was obtained from each participant before the experiment.

### Experimental Design and Materials

This was a within-subject designed study. The first factor was *TMS target*, i.e., the spTMS was targeted at the rVLPFC, the lVLPFC, and the vertex in three different blocks. The vertex was used as the TMS target in the sham condition to control the muscle twitches and noises generated during brain modulation. The second factor was *task*, i.e., the participants were randomly required to naturally attend to the picture or reappraise their emotion toward a less negative direction in each trial. There were 20 trials in each block (10 attend and 10 reappraise ones).

Sixty pictures depicting social exclusion scenarios were used to evoke negative emotional feelings. These pictures were selected from the image database of Social Inclusion and Exclusion in Young Asian Adults, which was developed by our laboratory for evoking self-relevant emotions in studies that explore emotional issues (Zheng et al., 2021). The pictures were assigned into six experimental conditions (attend-lVLPFC, attend-rVLPFC, attend-sham, reappraisal-lVLPFC, reappraisal-rVLPFC, and reappraisal-sham), with their arousal and valence ratings counterbalanced across different conditions. Before the formal experiment, three additional social exclusion pictures were used for practice. The stimuli were presented with E-prime 3.0 (Psychology Software Tools, Sharpsburg, United States). The size of each picture was 1200 pixels × 800. During the experiment, the images were presented in the center of an LCD monitor with a viewing angle of 3° × 3.5°. The screen was placed 60–70 cm from the participants’ eyes.

### Online Transcranial Magnetic Stimulation Protocol

**FIGURE 1:**
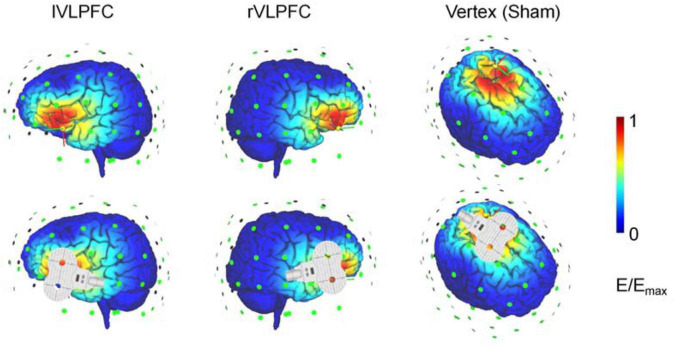
Illustration of the transcranial magnetic stimulation (TMS) electric fields of the three TMS conditions. The color represents the electric field strength, scaling from 0 (blue) to individual maximum (red).

Brain stimulation was delivered with a figure-eight-shaped coil (70 mm diameter) connected to a magnetic stimulator (M-100 Ultimate, Yingchi, Shenzhen, China). Single pulses were targeted in the left motor area to induce motor-evoked potentials (MEPs) with electrode slices fixed on the palm. Resting motor threshold (RMT) was defined as the lowest intensity evoking at least 5 MEP responses with amplitudes larger than 50 μV in 10 trials. The pulse intensity during the experiment was set as 120% of each participant’s RMT. Target regions were located based on the International 10/20 electroencephalogram system (the lVLPFC: F7; the rVLPFC: F8; vertex: Cz; and the left motor area: C3). The TMS-simulated electric field is illustrated in an adult brain model with SimNIBS (Figure 1; Thielscher et al., 2015). The participants wore earplugs and put their heads on a fixed chin rest to prevent head movements during the experiment. None of the 31 participants reported intolerable sensations or any adverse effects.

### Procedures

Before the experiment, the participants received brief instructions for the attending and reappraisal tasks. In the *Attend* condition, the participants were instructed to imagine themselves as the rejectee (the person highlighted with a red circle) in the picture. In the *Reappraisal* condition, the participants were instructed to not only experience the exclusion feelings of the rejectee but also reinterpret the situation to make themselves feel less negative. For instance, the participants were suggested to imagine that the group of individuals who were interacting with one another was talking about something that the participant (i.e., the person alone in the picture) was not interested in Zhao et al. (2021). In both the *Attend* and *Reappraisal* trials, the participants were required to verbalize their perception of pictures and emotional feelings, as well as their specific reinterpretations of the images in reappraisal trials. This oral procedure was designed to provide cognitive information and to ensure that the participants completed the task according to the attending or reappraisal requirements.

**FIGURE 2:**
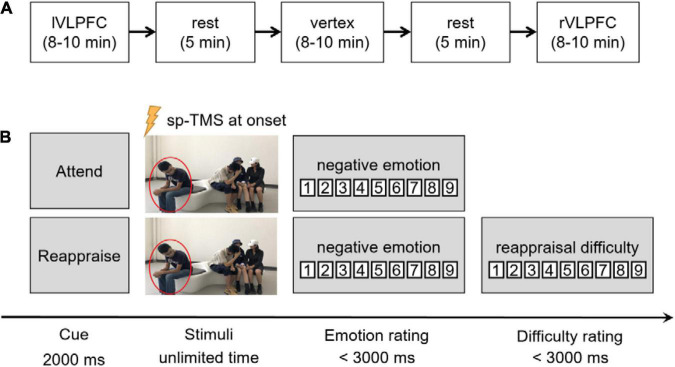
Experimental procedures. **(A)** Illustration of the experimental procedure. The order of the three blocks were counterbalanced across individuals. Here, we show an order of “the left VLPFC block” that came the first, followed by “the vertex block,” and “the right VLPFC block” came last. **(B)** Illustration of one trial in the task. After the cue of “Attend” or “Reappraise,” a social exclusion picture was presented until the participant finished his/her oral report. Single-pulse TMS (spTMS) was delivered at the onset of picture presentation. Negative emotion rating was required in each trial, and the rating of reappraisal difficulty was also required in the reappraisal condition.

The experiment began only after the participants understood the experimental requirements and could use the reappraisal strategies properly. The participants practiced in three trials (using another three images) before the formal experiment. Each participant needed to accomplish three blocks, each lasting for 8∼10 min, with different brain regions targeted by spTMS (Figure 2A). The sequence of the blocks was counterbalanced across individuals. A rest period of 5 min was inserted between neighboring blocks, during which the participants could close their eyes to have a rest. In each trial (Figure 2B), a task cue of “Attend” or “Reappraisal” with a single pulse was given at its onset. When the participants finished their oral reports, they needed to click the mouse to report their negative feelings (both in the *Attend* and *Reappraisal* trials) and reappraisal difficulty (only in the *Reappraisal* trials) in that trial on a 9-point scale (negative emotion: 1 = no negative feeling at all, 9 = extremely negative; reappraisal difficulty: 1 = extremely easy, 9 = extremely hard).

The oral reporting of the participants was recorded for *post hoc* analyses. The experimenters rated or examined the following indexes: total number of words, onset time of oral reporting, and verbal fluency. All the indexes were recognized and calculated using a python package for audio analysis, i.e., Librosa.^1^

### Statistical Analysis

The statistical analysis was performed using SPSS Statistics 26.0 (IBM, Somers, United States). Descriptive data were presented as mean M ± SD. Two-factors repeated-measures analyses of variances (ANOVAs) were performed on self-reported emotional ratings, reappraisal difficulty, and the indexes of the oral reporting. The Greenhouse-Geisser correction for the ANOVA tests was used whenever appropriate.

## Results

**FIGURE 3:**
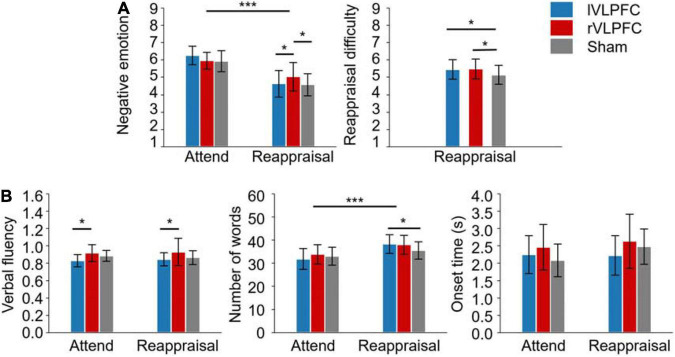
Results of the experiment. **(A)** Self-reported ratings of negative emotion and reappraisal difficulty. **(B)** Measures of oral reporting. The subplot in the right corner shows the onset time of oral reporting. Error bars represent ± SD. *p < 0.05; ***p < 0.001.

The descriptive statistics and the results of ANOVAs are reported in Table 1 and Illustrated in Figure 3.

**TABLE 1:** Descriptive statistics (M ± SD) and results of ANOVAs.

Descriptive			Main/Interaction effect	ANOVAs		
Group	Attend	Reappraisal		*F*	*p*	$\eta_{P}^{2}$
**Negative emotion**						
Sham	5.9 ± 0.2	4.6 ± 0.2	*TMS target*	30.1	<0.001***	0.501
lVLPFC	6.3 ± 0.2	4.6 ± 0.3	*task*	1.5	0.227	0.048
rVLPFC	6.0 ± 0.2	5.0 ± 0.3	*TMS target × task*	5.6	0.006**	0.158
**Reappraisal difficulty**						
Sham	/	5.1 ± 0.2	*TMS target*	/	/	/
lVLPFC	/	5.4 ± 0.2	*task*	3.6	0.034**	0.106
rVLPFC	/	5.5 ± 0.2	*TMS target × task*	/	/	/
**Verbal fluency**						
Sham	0.9 ± 0.0	0.9 ± 0.0	*TMS target*	0	0.94	0
lVLPFC	0.8 ± 0.0	0.8 ± 0.0	*task*	4.6	0.028**	0.132
rVLPFC	0.9 ± 0.0	0.9 ± 0.1	*TMS target × task*	0.4	0.637	0.012
**Number of words**						
Sham	5.1 ± 1.0	6.3 ± 1.1	*TMS target*	26.9	<0.001***	0.473
lVLPFC	7.5 ± 1.1	8.3 ± 1.4	*task*	1.4	0.247	0.046
rVLPFC	4.6 ± 1.1	6.3 ± 1.0	*TMS target × task*	4.2	0.020**	0.123
**Onset time (s)**						
Sham	2.1 ± 0.2	2.4 ± 0.2	*TMS target*	2.6	0.117	0.08
lVLPFC	2.3 ± 0.2	2.2 ± 0.2	*task*	2.2	0.125	0.067
rVLPFC	2.5 ± 0.2	2.6 ± 0.3	*TMS target × task*	2.2	0.117	0.069

**p < 0.01; ***p < 0.001. /, not applicable.

### Self-Reported Ratings

For the negative emotion rating, the main effect of *task* was significant (*F*(1,30) = 30.1, *p* < 0.001, $\eta_{\text{P}}^{2}$ = 0.501): the participants felt less negative in the reappraisal condition (4.8 ± 0.2) compared to the attending condition (6.1 ± 0.2). The main effect of *TMS target* was not significant (*F*(2, 60) = 1.5, *p* = 0.227, $\eta_{\text{P}}^{2}$ = 0.048). The interaction of *task × TMS target* was significant (*F*(2, 60) = 5.6, *p* = 0.006, $\eta_{\text{P}}^{2}$ 0.158; Figure 3A). Simple effect analysis reveals that the negative emotion differed significantly across TMS targets in the *Reappraisal* condition (*F*(2, 29) = 4.4, *p* < 0.05, $\eta_{\text{P}}^{2}$ 0.233): the participants felt more negative when their rVLPFC (5 ± 0.3) was temporally inhibited by TMS compared to the lVLPFC (4.6 ± 0.3, *t* = 2.246, *p* = 0.027) or sham (4.6 ± 0.2, *t* = 2.531, *p* = 0.013) condition. However, the effect of *TMS target* did not reach significance in the *Attend* condition (lVLPFC: 6.3 ± 0.2; rVLPFC: 6 ± 0.2; sham: 5.9 ± 0.2).

For the reappraisal difficulty, the one-way ANOVA found significant differences among the TMS targets (*F*(2, 60) = 3.6, *p* = 0.034, $\eta_{\text{P}}^{2}$ 0.106; Figure 3A): the participants had more reappraisal difficulty when their lVLPFC (5.4 ± 0.2, *t* = 2.191, *p* = 0.032) or rVLPFC (5.5 ± 0.2, *t* = 2.417, *p* = 0.019) was temporally inhibited by TMS compared to the sham condition (5.1 ± 0.2).

### Oral Reporting

For verbal fluency, the main effect of *TMS target* was significant (*F*(1, 30) = 4.6, *p* < 0.05, $\eta_{\text{P}}^{2}$ 0.132; Figure 3B): verbal fluency was lower in the lVLPFC condition (0.8 ± 0) than the in rVLPFC condition (0.9 ± .0, *t* = −3.01, *p* = 0.004). No other significant effects were observed (*p* > 0.05).

For the number of words, the main effect of *task* was significant (*F*(1, 30) = 26.9, *p* < 0.001, $\eta_{\text{P}}^{2}$ 0.473): the participants said more words in the *Reappraisal* condition (37.3 ± 1.2) than in the *Attend* condition (32.9 ± 1.4). The interaction of *task × TMS target* was also significant (*F*(2, 60) = 4.2, *p* < 0.05, $\eta_{\text{P}}^{2}$ = 0.123; Figure 3B). The simple effect analysis reveals that the number of words in the lVLPFC block (38.3 ± 1.4) was larger than that in the sham block (35.6 ± 1.4, *t* = 2.269, *p* = 0.025) during the reappraisal but not during the attending task(*t* = −0.942, *p* = 0.348).

No significant effect was found in the onset time of oral reporting (*p* > 0.05).

## Discussion

This study conducted an online spTMS to investigate the separate roles of the left and right VLPFC in reappraisal. It was found that inhibiting either side of the VLPFC could always increase the difficulty of reappraisal. Meanwhile, the reappraisal outcome indexed by negative emotion rating significantly deteriorated when the rVLPFC was disturbed by spTMS, while the measures of oral reporting suggested that inhibited lVLPFC resulted in increased number of words during reappraisal and decreased verbal fluency irrespective of attending or reappraisal task. These findings indicated distinct roles of the left and right VLPFC in cognitive reappraisal.

The results showed deteriorated reappraisal outcome and increased reappraisal difficulty caused by the interference over the rVLPFC. This finding is in line with the hypothesis on the essential role of the rVLPFC in reappraisal implementation. Many studies have suggested the association between activation of rVLPFC and self-reported negative emotions (Eisenberger et al., 2003; Phan et al., 2005; Masten et al., 2009), as well as reappraisal success (Wager et al., 2008). Also, a previous study in our laboratory has provided causal evidence to support the critical role of the rVLPFC in reappraisal outcome (He Z. et al., 2018; He et al., 2020a,b; Zhao J. et al., 2021; Li et al., 2022). It is well-known that the rVLPFC is an essential region in the inhibition process (Aron et al., 2004; Leung and Cai, 2007; Levy and Wagner, 2011; Rae et al., 2015; Apšvalka et al., 2022), which is usually observed in the Go/No-go, Think/No-think, and Stop Signal tasks (refer to meta-analysis by Levy and Wagner (2011), Apšvalka et al. (2022)). For instance, Guo et al. (2018) that found recruitment of the rVLPFC is indispensable for thought inhibition in the No-think condition of the Think/No-think task. Additionally, Riva et al. (2015) conducted anodal transcranial direct current stimulation (tDCS) over the rVLPFC and observed inhibited aggressive behaviors when participants faced negative situations. Likewise, Kaladjian et al. (2007, 2011) proved that abnormal activation in the rVLPFC resulted in reduced inhibition of impulsiveness in patients with schizophrenia. Moreover, Wang et al. (2018) reported that right lateralization of the frontal alpha asymmetry (FAA), which has a neural source at the VLPFC, could predict the habitual use of reappraisal. Since the alpha oscillation is considered to be a signal of inhibition (Klimesch, 2012), the right lateralization of the FAA suggested the inhibition role of reappraisal mainly located on the right part of the VLPFC. Although these studies have suggested that the rVLPFC is associated with the inhibitory function during emotion regulation, straightforward evidence for the inhibitory role of the rVLPFC during reappraisal is still lacking and requires further examination.

The most important and novel finding of this study was the reduced verbal fluency accompanied by increased spoken words in the lVLPFC-interfered condition. This finding is in line with previous studies indicating the linguistic role of the lVLPFC (Winhuisen et al., 2005; Fu et al., 2018). Beyond these findings, this study conducted oral reporting to measure the semantic and spoken processes of reappraisal, which provided a direct observation of the verbal processing in reappraisal and gave straightforward evidence for the linguistic role of the lVLPFC in reappraisal (Ochsner et al., 2012; Buhle et al., 2014; Morawetz et al., 2016; Keller et al., 2021; Cao et al., 2022). Neuroimaging studies provided abundant evidence to support the important role of the lVLPFC in semantic representation and selection (Badre and Wagner, 2007; Nagel et al., 2008; Souza et al., 2009; Conner et al., 2019; Zhang et al., 2022). Since the implementation of reappraisal needs to generate and select proper appraisals for emotional situation (Buhle et al., 2014; Kohn et al., 2014; Berboth and Morawetz, 2021), spTMS over the lVLPFC disturbed the semantic process and made reappraisal quite difficult. Furthermore, the lVLPFC is critical not only for semantic processing but also for language production (i.e., spoken processing; Friederici, 2011). For example, it was found that patients with stroke and apraxia of speech had abnormal connectivity between the lVLPFC and the premotor cortex (New et al., 2015). Besides, patients with schizophrenia and elevated cytokine performed significantly worse on verbal fluency with decreased volume in Broca’s area (overlapping with the lVLPFC; Fillman et al., 2016). Thus, the interference over the lVLPFC could also negatively impact the syllabifying and articulation processes in our oral emotion regulation task. Taken together, this study found that the lVLPFC is responsible for both the internal (semantic processing) and external (spoken language) language processes during cognitive reappraisal.

In line with the current findings, we propose that there is a functional asymmetry of the bilateral VLPFC in reappraisal. On the one hand, the rVLPFC may be recruited for the direct emotion processing of cognitive inhibition. When receiving negative emotional stimuli, the rVLPFC quickly initiates the inhibition process by orienting attention from the goal-irrelative information and blocking inappropriate negative emotions and thoughts (Ochsner et al., 2012; Hanlon et al., 2017). On the other hand, the lVLPFC works to generate, represent, and select semantic meanings during the implementation of reappraisal (Buhle et al., 2014; Berboth and Morawetz, 2021). The effectiveness of lVLPFC’s semantic processing is directly related to the difficulty of emotion regulation (Johnstone et al., 2007; Giuliani et al., 2020; Keller et al., 2021). Finally, it should be noted that the TMS was targeted to the vertex in the sham condition. Some studies have demonstrated the involvement of the parietal cortex (including the vertex) during emotion regulation. Therefore, using the vertex as the controlled brain area might have introduced some confounding effects in our results. Future studies are needed to verify the current findings using another irrelevant brain region in the controlled condition.

In conclusion, we found that the left and right sides of the VLPFC differently impact verbal and affective outcomes during cognitive reappraisal, which supported the hypothesized dissociation roles of bilateral VLPFC in reappraisal. In particular, while the rVLPFC plays an inhibitory role in downregulation of negative emotions, the lVLPFC mainly serves as a semantic processor that generates and select appropriate appraisals. These findings highlight the distinct roles of the left and right VLPFC in reappraisal and deepen our understanding of the neurocognitive mechanism of emotion regulation.

## Data Availability Statement

The original contributions presented in this study are included in the article/supplementary material, further inquiries can be directed to the corresponding author.

## Ethics Statement

The studies involving human participants were reviewed and approved by the Ethical Committee of Shenzhen University. The patients/participants provided their written informed consent to participate in this study.

## Author Contributions

DZ and SC designed the research. SC, XQ, and FX performed the experiments. DZ, SC, and XQ analyzed the data. DZ, SC, SL, and LM wrote the article. All authors contributed to the article and approved the submitted version.

## Conflict of Interest

FX was employed by Shenzhen Yingchi Technology Co., Ltd. The remaining authors declare that the research was conducted in the absence of any commercial or financial relationships that could be construed as a potential conflict of interest.

## Publisher’s Note

All claims expressed in this article are solely those of the authors and do not necessarily represent those of their affiliated organizations, or those of the publisher, the editors and the reviewers. Any product that may be evaluated in this article, or claim that may be made by its manufacturer, is not guaranteed or endorsed by the publisher.

## Footnotes

1
https://librosa.org/

## References

[B1] Apšvalka D. Ferreira C. S. Schmitz T. W. Rowe J. B. Anderson M. C. (2022). Dynamic targeting enables domain-general inhibitory control over action and thought by the prefrontal cortex. *Nat. Commun.* 13:274. 10.1038/s41467-021-27926-w 35022447 PMC8755760

[B2] Aron A. R. Robbins T. W. Poldrack R. A. (2004). Inhibition and the right inferior frontal cortex. *Trends Cogn. Sci.* 8 170–177. 10.1016/j.tics.2004.02.010 15050513

[B3] Badre D. Wagner A. D. (2007). Left ventrolateral prefrontal cortex and the cognitive control of memory. *Neuropsychologia* 45 2883–2901. 10.1016/j.neuropsychologia.2007.06.015 17675110

[B4] Banjac S. Roger E. Pichat C. Cousin E. Mosca C. Lamalle L. (2021). Reconfiguration dynamics of a language-and-memory network in healthy participants and patients with temporal lobe epilepsy. *Neuroimage Clin.* 31:102702. 10.1016/j.nicl.2021.102702 34090125 PMC8186554

[B5] Berboth S. Morawetz C. (2021). Amygdala-prefrontal connectivity during emotion regulation: A meta-analysis of psychophysiological interactions. *Neuropsychologia* 153:107767. 10.1016/j.neuropsychologia.2021.107767 33516732

[B6] Billingsley R. L. McAndrews M. P. Crawley A. P. Mikulis D. J. (2001). Functional MRI of phonological and semantic processing in temporal lobe epilepsy. *Brain* 124(Pt 6), 1218–1227. 10.1093/brain/124.6.1218 11353737

[B7] Buhle J. T. Silvers J. A. Wager T. D. Lopez R. Onyemekwu C. Kober H. (2014). Cognitive reappraisal of emotion: a meta-analysis of human neuroimaging studies. *Cereb. Cortex* 24 2981–2990. 10.1093/cercor/bht154 23765157 PMC4193464

[B8] Cao D. Qian Z. Tang Y. Wang J. Jiang T. Li Y. (2022). Neural indicator of positive reappraisal: a TMS-EEG study over the left VLPFC. *J. Affect Disord.* 300 418–429. 10.1016/j.jad.2021.12.136 34986377

[B9] Cho S. Metcalfe A. W. Young C. B. Ryali S. Geary D. C. Menon V. (2012). Hippocampal-prefrontal engagement and dynamic causal interactions in the maturation of children’s fact retrieval. *J. Cogn. Neurosci.* 24 1849–1866. 10.1162/jocn_a_00246 22621262 PMC3462165

[B10] Conner C. R. Kadipasaoglu C. M. Shouval H. Z. Hickok G. Tandon N. (2019). Network dynamics of Broca’s area during word selection. *PLoS One* 14:e0225756. 10.1371/journal.pone.0225756 31860640 PMC6924671

[B11] Eisenberger N. I. Lieberman M. D. Williams K. D. (2003). Does rejection hurt? An FMRI study of social exclusion. *Science* 302 290–292. 10.1126/science.1089134 14551436

[B12] Erk S. von Kalckreuth A. Walter H. (2010). Neural long-term effects of emotion regulation on episodic memory processes. *Neuropsychologia* 48 989–996. 10.1016/j.neuropsychologia.2009.11.022 19945471

[B13] Fillman S. G. Weickert T. W. Lenroot R. K. Catts S. V. Bruggemann J. M. Catts V. S. (2016). Elevated peripheral cytokines characterize a subgroup of people with schizophrenia displaying poor verbal fluency and reduced Broca’s area volume. *Mol. Psychiatry* 21 1090–1098. 10.1038/mp.2015.90 26194183 PMC4960447

[B14] Friederici A. D. (2011). The brain basis of language processing: from structure to function. *Physiol. Rev.* 91 1357–1392. 10.1152/physrev.00006.2011 22013214

[B15] Fu L. Xiang D. Xiao J. Yao L. Wang Y. Xiao L. (2018). Reduced Prefrontal Activation During the Tower of London and Verbal Fluency Task in Patients With Bipolar Depression: a Multi-Channel NIRS Study. *Front. Psychiatry* 9:214. 10.3389/fpsyt.2018.00214 29892235 PMC5985469

[B16] Giuliani N. R. Cosme D. Merchant J. S. Dirks B. Berkman E. T. (2020). Brain Activity Associated With Regulating Food Cravings Predicts Changes in Self-Reported Food Craving and Consumption Over Time [Article]. *Front. Hum. Neurosci.* 14:577669. 10.3389/fnhum.2020.577669 33281580 PMC7689031

[B17] Guo Y. Schmitz T. W. Mur M. Ferreira C. S. Anderson M. C. (2018). A supramodal role of the basal ganglia in memory and motor inhibition: Meta-analytic evidence. *Neuropsychologia* 108 117–134. 10.1016/j.neuropsychologia.2017.11.033 29199109 PMC5759998

[B18] Hanlon F. M. Dodd A. B. Ling J. M. Bustillo J. R. Abbott C. C. Mayer A. R. (2017). From Behavioral Facilitation to Inhibition: the Neuronal Correlates of the Orienting and Reorienting of Auditory Attention. *Front. Hum. Neurosci.* 11:293. 10.3389/fnhum.2017.00293 28634448 PMC5459904

[B19] He X. Bassett D. S. Chaitanya G. Sperling M. R. Kozlowski L. Tracy J. I. (2018). Disrupted dynamic network reconfiguration of the language system in temporal lobe epilepsy. *Brain* 141 1375–1389. 10.1093/brain/awy042 29554279 PMC13045878

[B20] He Z. Lin Y. Xia L. Liu Z. Zhang D. Elliott R. (2018). Critical role of the right VLPFC in emotional regulation of social exclusion: a tDCS study. *Soc. Cogn. Affect Neurosci.* 13 357–366. 10.1093/scan/nsy026 29618116 PMC5928413

[B21] He Z. Liu Z. Zhao J. Elliott R. Zhang D. (2020a). Improving emotion regulation of social exclusion in depression-prone individuals: a tDCS study targeting right VLPFC. *Psychol. Med.* 50 2768–2779. 10.1017/S0033291719002915 31615594

[B22] He Z. Zhao J. Shen J. Muhlert N. Elliott R. Zhang D. (2020b). The right VLPFC and downregulation of social pain: a TMS study. *Hum. Brain Mapp.* 41 1362–1371. 10.1002/hbm.24881 31789480 PMC7267938

[B23] Johnstone T. van Reekum C. M. Urry H. L. Kalin N. H. Davidson R. J. (2007). Failure to regulate: counterproductive recruitment of top-down prefrontal-subcortical circuitry in major depression. *J. Neurosci.* 27 8877–8884. 10.1523/JNEUROSCI.2063-07.2007 17699669 PMC6672169

[B24] Kaladjian A. Jeanningros R. Azorin J. M. Anton J. L. Mazzola-Pomietto P. (2011). Impulsivity and neural correlates of response inhibition in schizophrenia. *Psychol. Med.* 41 291–299. 10.1017/s0033291710000796 20406530

[B25] Kaladjian A. Jeanningros R. Azorin J. M. Grimault S. Anton J. L. Mazzola-Pomietto P. (2007). Blunted activation in right ventrolateral prefrontal cortex during motor response inhibition in schizophrenia. *Schizophr. Res.* 97 184–193. 10.1016/j.schres.2007.07.033 17855057

[B26] Keller M. Zweerings J. Klasen M. Zvyagintsev M. Iglesias J. Mendoza Quiñones R. (2021). fMRI Neurofeedback-Enhanced Cognitive Reappraisal Training in Depression: a Double-Blind Comparison of Left and Right vlPFC Regulation. *Front. Psychiatry* 12:715898. 10.3389/fpsyt.2021.715898 34497546 PMC8419460

[B27] Klimesch W. (2012). α-band oscillations, attention, and controlled access to stored information. *Trends Cogn. Sci.* 16 606–617. 10.1016/j.tics.2012.10.007 23141428 PMC3507158

[B28] Kohn N. Eickhoff S. B. Scheller M. Laird A. R. Fox P. T. Habel U. (2014). Neural network of cognitive emotion regulation–an ALE meta-analysis and MACM analysis. *Neuroimage* 87 345–355. 10.1016/j.neuroimage.2013.11.001 24220041 PMC4801480

[B29] Leung H. C. Cai W. (2007). Common and differential ventrolateral prefrontal activity during inhibition of hand and eye movements. *J. Neurosci.* 27 9893–9900. 10.1523/jneurosci.2837-07.2007 17855604 PMC6672638

[B30] Levy B. J. Wagner A. D. (2011). Cognitive control and right ventrolateral prefrontal cortex: reflexive reorienting, motor inhibition, and action updating. *Ann. N. Y. Acad. Sci.* 1224 40–62. 10.1111/j.1749-6632.2011.05958.x 21486295 PMC3079823

[B31] Li S. Xie H. Zheng Z. Chen W. Xu F. Hu X. (2022). The causal role of the bilateral ventrolateral prefrontal cortices on emotion regulation of social feedback. *Hum. Brain Mapp*. 2022:25824. 10.1002/hbm.25824 35261115 PMC9120569

[B32] Marini A. Urgesi C. (2012). Please get to the point! A cortical correlate of linguistic informativeness. *J. Cogn. Neurosci.* 24 2211–2222. 10.1162/jocn_a_00283 22905815

[B33] Masten C. L. Eisenberger N. I. Borofsky L. A. Pfeifer J. H. McNealy K. Mazziotta J. C. (2009). Neural correlates of social exclusion during adolescence: understanding the distress of peer rejection. *Soc. Cogn. Affect Neurosci.* 4 143–157. 10.1093/scan/nsp007 19470528 PMC2686232

[B34] McRae K. Gross J. J. (2020). Emotion regulation. *Emotion* 20 1–9. 10.1037/emo0000703 31961170

[B35] Morawetz C. Bode S. Baudewig J. Jacobs A. M. Heekeren H. R. (2016). Neural representation of emotion regulation goals. *Hum. Brain Mapp.* 37 600–620. 10.1002/hbm.23053 26537018 PMC6867353

[B36] Morawetz C. Bode S. Derntl B. Heekeren H. R. (2017). The effect of strategies, goals and stimulus material on the neural mechanisms of emotion regulation: a meta-analysis of fMRI studies. *Neurosci. Biobehav. Rev.* 72 111–128. 10.1016/j.neubiorev.2016.11.014 27894828

[B37] Nagel I. E. Schumacher E. H. Goebel R. D’Esposito M. (2008). Functional MRI investigation of verbal selection mechanisms in lateral prefrontal cortex. *Neuroimage* 43 801–807. 10.1016/j.neuroimage.2008.07.017 18692142 PMC2612124

[B38] New A. B. Robin D. A. Parkinson A. L. Duffy J. R. McNeil M. R. Piguet O. (2015). Altered resting-state network connectivity in stroke patients with and without apraxia of speech. *Neuroimage Clin.* 8 429–439. 10.1016/j.nicl.2015.03.013 26106568 PMC4473263

[B39] Ochsner K. N. Silvers J. A. Buhle J. T. (2012). Functional imaging studies of emotion regulation: a synthetic review and evolving model of the cognitive control of emotion. *Ann. N. Y. Acad. Sci.* 1251 E1–E24. 10.1111/j.1749-6632.2012.06751.x 23025352 PMC4133790

[B40] Ortner C. N. Ste Marie M. Corno D. (2016). Cognitive Costs of Reappraisal Depend on Both Emotional Stimulus Intensity and Individual Differences in Habitual Reappraisal. *PLoS One* 11:e0167253. 10.1371/journal.pone.0167253 27936022 PMC5147884

[B41] Phan K. L. Fitzgerald D. A. Nathan P. J. Moore G. J. Uhde T. W. Tancer M. E. (2005). Neural substrates for voluntary suppression of negative affect: a functional magnetic resonance imaging study. *Biol. Psychiatry* 57 210–219. 10.1016/j.biopsych.2004.10.030 15691521

[B42] Price C. J. Crinion J. T. Macsweeney M. (2011). A Generative Model of Speech Production in Broca’s and Wernicke’s Areas. *Front. Psychol.* 2:237. 10.3389/fpsyg.2011.00237 21954392 PMC3174393

[B43] Rae C. L. Hughes L. E. Anderson M. C. Rowe J. B. (2015). The prefrontal cortex achieves inhibitory control by facilitating subcortical motor pathway connectivity. *J. Neurosci.* 35 786–794. 10.1523/jneurosci.3093-13.2015 25589771 PMC4293423

[B44] Riva P. Romero Lauro L. J. DeWall C. N. Chester D. S. Bushman B. J. (2015). Reducing aggressive responses to social exclusion using transcranial direct current stimulation. *Soc. Cogn. Affect Neurosci.* 10 352–356. 10.1093/scan/nsu053 24748546 PMC4350477

[B45] Souza M. J. Donohue S. E. Bunge S. A. (2009). Controlled retrieval and selection of action-relevant knowledge mediated by partially overlapping regions in left ventrolateral prefrontal cortex. *Neuroimage* 46 299–307. 10.1016/j.neuroimage.2009.01.046 19457379 PMC3090080

[B46] Spielberger C. D. Gorsuch R. L. Lushene R. E. Vagg P. R. Jacobs G. A. (1983). *Manual for the State-Trait Anxiety Inventory (Form Y1 – Y2).* Palo Alto: Consulting Psychologist Press.

[B47] Sylvester C. M. Barch D. M. Harms M. P. Belden A. C. Oakberg T. J. Gold A. L. (2016). Early Childhood Behavioral Inhibition Predicts Cortical Thickness in Adulthood. *J. Am. Acad. Child Adolesc. Psychiatry* 55 122.e–129.e. 10.1016/j.jaac.2015.11.007 26802779 PMC4724382

[B48] Thielscher A. Antunes A. Saturnino G. B. (2015). Field modeling for transcranial magnetic stimulation: a useful tool to understand the physiological effects of TMS? *Annu. Int. Conf. IEEE Eng. Med. Biol. Soc.* 2015 222–225. 10.1109/embc.2015.7318340 26736240

[B49] Troy A. S. Shallcross A. J. Brunner A. Friedman R. Jones M. C. (2018). Cognitive reappraisal and acceptance: effects on emotion, physiology, and perceived cognitive costs. *Emotion* 18 58–74. 10.1037/emo0000371 29154585 PMC6188704

[B50] Urgesi C. Mattiassi A. D. Buiatti T. Marini A. (2016). Tell it to a child! A brain stimulation study of the role of left inferior frontal gyrus in emotion regulation during storytelling. *Neuroimage* 136 26–36. 10.1016/j.neuroimage.2016.05.039 27188219

[B51] Wager T. D. Davidson M. L. Hughes B. L. Lindquist M. A. Ochsner K. N. (2008). Prefrontal-subcortical pathways mediating successful emotion regulation. *Neuron* 59 1037–1050. 10.1016/j.neuron.2008.09.006 18817740 PMC2742320

[B52] Walsh T. (1983). Voice-onset time as a clue to the nature of Broca speech errors. *Brain Lang.* 19 357–363. 10.1016/0093-934x(83)90077-9 6883078

[B53] Wang Y. Lu J. Gu C. Hu B. (2018). Mapping the frontal alpha asymmetry indicators of habitual emotion regulation: a data-driven approach. *Neuroreport* 29 1288–1292. 10.1097/wnr.0000000000001109 30095582

[B54] Winhuisen L. Thiel A. Schumacher B. Kessler J. Rudolf J. Haupt W. F. (2005). Role of the contralateral inferior frontal gyrus in recovery of language function in poststroke aphasia: a combined repetitive transcranial magnetic stimulation and positron emission tomography study. *Stroke* 36 1759–1763. 10.1161/01.STR.0000174487.81126.ef 16020770

[B55] Zhang M. Nathaniel U. Savill N. Smallwood J. Jefferies E. (2022). Intrinsic connectivity of left ventrolateral prefrontal cortex predicts individual differences in controlled semantic retrieval. *Neuroimage* 246:118760. 10.1016/j.neuroimage.2021.118760 34875381 PMC8784820

[B56] Zhang Q. Yu B. Zhang J. Jin Z. Li L. (2018). Probing the Timing Recruitment of Broca’s Area in Speech Production for Mandarin Chinese: a TMS Study. *Front. Hum. Neurosci.* 12:133. 10.3389/fnhum.2018.00133 29692715 PMC5902490

[B57] Zhao J. Mo L. Bi R. He Z. Chen Y. Xu F. (2021). The VLPFC versus the DLPFC in Downregulating Social Pain Using Reappraisal and Distraction Strategies. *J. Neurosci.* 41 1331–1339. 10.1523/JNEUROSCI.1906-20.2020 33443069 PMC7888223

[B58] Zhao Z. Liu Y. Zhang J. Lu J. Wu J. (2021). Where is the speech production area? Evidence from direct cortical electrical stimulation mapping. *Brain* 144:e61. 10.1093/brain/awab178 33978731 PMC8370392

[B59] Zheng Z. Li S. Mo L. Chen W. Zhang D. (2021). ISIEA: an image database of social inclusion and exclusion in young Asian adults. *Behav. Res. Methods* 2021:1736. 10.3758/s13428-021-01736-w 34918228 PMC9579065

[B60] Zung W. W. Richards C. B. Short M. J. (1965). Self-rating depression scale in an outpatient clinic. Further validation of the SDS. *Arch. Gen. Psychiatry* 13 508–515. 10.1001/archpsyc.1965.01730060026004 4378854

